# Characterization and Gelling Potential of Macroalgae Extracts Obtained Through Eco-Friendly Technologies for Food-Grade Gelled Matrices

**DOI:** 10.3390/gels11040290

**Published:** 2025-04-15

**Authors:** Filipe Vinagre, Maria João Alegria, Andreia Sousa Ferreira, Cláudia Nunes, Maria Cristiana Nunes, Anabela Raymundo

**Affiliations:** 1 LEAF—Linking Landscape, Environment, Agriculture and Food Research Center, Associate Laboratory TERRA, School of Agriculture, University of Lisbon, Tapada da Ajuda, 1349-017 Lisbon, Portugal; fafvinagre@gmail.com (F.V.); maria.alegria@sumolcompal.pt (M.J.A.); anabraymundo@isa.ulisboa.pt (A.R.); 2 SUMOL + COMPAL Marcas S.A., 2780-179 Carnaxide, Portugal; 3 LAQV-REQUIMTE, Department of Chemistry, University of Aveiro, Campus Universitário de Santiago, 3810-193 Aveiro, Portugal; a39493@ua.pt; 4 CICECO—Aveiro Institute of Materials, Department of Materials and Ceramic Engineering, University of Aveiro, Campus Universitário de Santiago, 3810-193 Aveiro, Portugal; claudianunes@ua.pt

**Keywords:** macroalgae, hydrocolloids, green extraction, rheological properties, food hydrocolloids

## Abstract

The growing demand for sustainable and nutrient-rich food sources has positioned macroalgae as a promising alternative for food product development. This study investigates the extraction and characterization of hydrocolloids from three red macroalgae species (*Chondrus crispus*, *Gracilaria gracilis*, and *Gelidium corneum*) using water bath (WB), ultrasound (US), and hybrid ultrasound–water bath (USWB) treatments for 45 and 60 min. The physicochemical properties of the extracts, including rheological behavior, particle size distribution, antioxidant activity, and texture, were assessed. The results show that *C. crispus* produced the firmest gels due to its high carrageenan content, with WB and USWB treatments yielding the most stable gel structures. In contrast, *G. gracilis* and *G. corneum* exhibited lower gel strength, consistent with their agar composition. WB-treated samples demonstrated superior antioxidant retention, while US treatment was more effective in preserving color stability. The findings highlight macroalgae as a viable and sustainable alternative to conventional hydrocolloids, reinforcing their potential as natural gelling agents, thickeners, and stabilizers for the food and pharmaceutical industries. This study provides a comparative evaluation of WB, US, and USWB extraction methods, offering insights into optimizing hydrocolloid extraction for enhanced functionality and sustainability.

## 1. Introduction

The global population is projected to surpass 9.7 billion by 2050, intensifying the strain on food production systems [1]. Traditional agriculture faces growing constraints by limited land and water resources, while their environmental footprint remains a major concern. To address these pressing challenges, the search for alternative food sources that are both sustainable and nutrient-dense is imperative. Seaweeds, or macroalgae, present a highly promising solution. Depending on the species, they can be rich in proteins, polysaccharides, vitamins, and minerals [2,3,4]. Among them, *Chondrus crispus* (Irish moss) is notable for its kappa-carrageenan content, a compound widely valued for its gelling and thickening properties in food products such as dairy alternatives and sauces [5]. Beyond their nutritional value, seaweeds contribute significantly to reducing environmental impact. For example, wild seaweeds absorb substantial amounts of carbon oxide (CO_2_) from the atmosphere, playing a vital role in climate change mitigation [6]. Additionally, seaweed farming requires neither freshwater nor arable land, making it an environmentally sustainable option for large-scale production [7]. In addition to carbon sequestration, seaweed farming offers critical environmental benefits. Macroalgae cultivation helps counteract ocean acidification by absorbing excess CO_2_ from seawater, thereby raising pH levels and creating a more hospitable environment for marine life [8]. Furthermore, seaweeds actively contribute to nutrient cycling by absorbing nitrogen and phosphorus from surrounding waters, which helps reduce the occurrence of harmful algal blooms [9]. Integrating seaweed farming into existing aquaculture systems amplifies these environmental advantages, supporting the development of more sustainable and resilient coastal ecosystems. However, the large-scale seaweed processing faces challenges related to seasonal availability, post-harvest handling, and variability in bioactive compound content [10]. Although macroalgae cultivation requires minimal freshwater or arable land, processing costs are largely influenced by extraction efficiency and hydrocolloid recovery optimization. Enhancing these methods, as explored in this study, is essential for improving economic feasibility and establishing seaweed-derived hydrocolloids as a commercially viable alternative [11]. Additionally, seaweeds provide numerous health benefits due to their high content of bioactive compounds, such as antioxidants, sulfated polysaccharides, and dietary fibers, which have been linked to anti-inflammatory, anti-cancer, and metabolic health benefits [12]. However, they can also accumulate heavy metals and other environmental contaminants from polluted waters, presenting potential safety risks to consumers. Therefore, careful sourcing and rigorous quality control measures are crucial to ensuring product safety and regulatory compliance [13,14]. Traditional methods for extracting carrageenan from seaweeds are often harsh and energy intensive [15]. The growing demand for sustainable and efficient extraction methods has spurred the development of techniques such as ultrasound-assisted extraction (UAE) and water bath extraction. These methods are considered more eco-friendly than conventional solvent extraction due to their lower energy consumption, reduced use of solvents, and shorter processing times. UAE, in particular, is notable for its ability to enhance mass transfer and disrupt cellular structures, facilitating the efficient release of bioactive compounds without the need for excessive heating or harsh solvents [16]. This technique not only improves extraction yields, but also preserves the structural integrity of heat-sensitive compounds, such as phenolic compounds [15,17].

Water bath extraction, while less mechanically disruptive than UAE, provides a controlled environment for extracting hydrocolloids like carrageenan and agar. Its temperature-controlled conditions maintain the bioactivity of compounds while consuming less energy compared to traditional boiling or reflux methods [18]. Both techniques align with green chemistry principles by generating minimal chemical waste and using renewable water sources instead of organic solvents. These eco-friendly methods enhance the yield and quality of carrageenan while meeting consumer demand for “clean label” food ingredients [19]. Given the growing industrial emphasis on minimizing chemical usage and energy consumption, optimizing these extraction techniques could support the large-scale adoption of hydrocolloids in functional food and pharmaceutical applications.

Recent studies have confirmed the effectiveness of these methods, revealing that carrageenan extracted through eco-friendly processes retains its gelling properties, making it highly suitable for food applications [20].

Despite their widespread applications in the food, pharmaceutical, and biotechnology industries, hydrocolloid extraction remains challenging due to high energy consumption and chemical use [15,21]. To overcome these challenges, novel and eco-friendly extraction methods, such as ultrasound-assisted (UAE) and microwave-assisted (MAE) extractions, have been investigated for their potential to improve hydrocolloid yield while minimizing environmental impact [22]. However, despite significant advancements in alternative extraction techniques, comparative studies specifically assessing the efficiency of water bath (WB), ultrasound (US), and hybrid ultrasound–water bath (USWB) extraction in red macroalgae species—such as *Chondrus crispus*, *Gracilaria gracilis*, and *Gelidium corneum*—remain scare [15,23]. Recent reviews emphasize the importance of optimizing extraction techniques to preserve the functional properties of hydrocolloids while maximizing their yield [15,21]. The present study systematically compares WB, US, and USWB extraction across three red macroalgae species, assessing their impact on hydrocolloid properties and bioactivity. Unlike previous research, it provides insights that could inform future advancements in sustainable hydrocolloid production for food, pharmaceutical, and biomedical applications. This study aims to address this gap by systematically evaluating the impact of water bath (WB), ultrasound (US), and hybrid ultrasound–water bath (USWB) extraction techniques on the rheological properties, polysaccharides content and composition, antioxidant activity, and sensory characteristics of seaweed-derived gels. The novelty of this research lies in its comprehensive approach, integrating advanced extraction techniques with detailed physicochemical and sensory analyses. The findings are expected to provide valuable insights into the potential industrial applications of these seaweed species, particularly in the food and pharmaceutical sectors.

## 2. Results and Discussion

### 2.1. Nutritional Characterization

The selected macroalgae are known to contain structural polysaccharides, such as cellulose and agarose, which contribute to their textural properties and potential application in hydrocolloid extraction. The data summarized in the Seaweed Samples section of the Materials and Methods indicate that *C. crispus* and *G. gracilis* generally exhibit higher protein content compared to *G. corneum*, while the latter tends to contain slightly more lipids. These species also provide essential amino acids and only trace amounts of polyunsaturated fatty acids, including omega-3 [23,24,25,26].

The higher protein content in *C. crispus* and *G. gracilis* underscores their potential as alternative protein sources in food formulations. Red macroalgae are particularly rich in mycosporine-like amino acids, which confer antioxidant and photoprotective benefits [27,28] Meanwhile, the slightly higher lipid content in *G. corneum* may enhance the bioavailability of lipophilic compounds in nutraceutical applications [29]. These findings are consistent with previous studies reporting red macroalgae protein levels of 10–30% dry weight, influenced by seasonal and geographical factors [30,31] Species selection is a key determinant of industrial applications, with protein-rich species like *C. crispus* and *G. gracilis* being well-suited for functional food development, while the lipid profile of *G. corneum* may offer advantages for bioactive ingredient delivery [32].

### 2.2. Particle Size Distribution of Seaweed Powders

Table 1 presents the particle size distribution of the seaweed powders, a critical factor in hydrocolloid extraction. Particle size influences solubility, mass transfer, and hydrocolloid yield, making it a key parameter for optimizing extraction efficiency.

**Figure 1:**
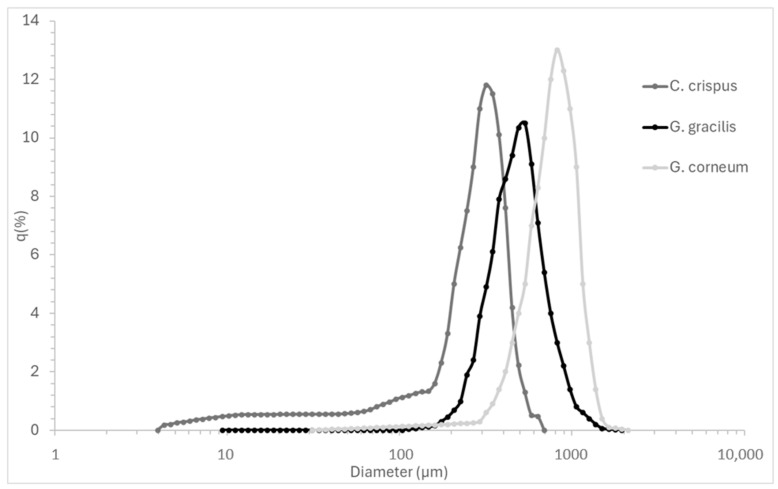
Particle size distribution curves (q%) of *Chondrus crispus*, *Gelidium corneum*, and *Gracilaria gracilis* powders, illustrating the frequency and dispersion of particle sizes.

Figure 1 complements the numerical data by visually depicting the particle size distribution curves, highlighting the size dispersion and polydispersity of each seaweed powder.

Particle size directly influences hydrocolloid extraction efficiency by affecting mass transfer, solubility, and hydrocolloid release kinetics. Smaller particles provide a higher surface-area-to-volume ratio, enhancing solvent penetration and extraction yields [33]. This is particularly relevant in industrial-scale processing, where optimizing particle size can improve extraction rates while minimizing processing time and energy costs [34].

Although smaller particles can theoretically agglomerate and hinder solvent penetration due to increased cohesive forces [17], this effect was not observed in this study. In fact, *C. crispus*, which exhibited the smallest particle size, achieved the most effective extraction in terms of gelation, indicating that particle agglomeration did not hinder hydrocolloid recovery under the tested conditions.

*G. gracilis* exhibited an intermediate D50 value (0.550 mm), with a relatively high D90 (1.108 mm), suggesting the presence of larger aggregates. The mean particle size (0.635 mm) and broad standard deviation (±0.353 mm) indicate variability in particle size distribution, which may lead to inconsistencies in extraction efficiency. Larger particle variability could require additional milling or processing adjustments in industrial settings to ensure homogeneous hydrocolloid recovery [35] *C. crispus* showed the finest particle size distribution, with the smallest D50 value (0.329 mm), D10 (0.052 mm), and D90 (0.568 mm) values. The smaller mean particle size (0.330 mm) and narrower standard deviation (±0.193 mm) suggest a more uniform particle size distribution. This consistency is likely to promote more efficient extraction, as finer and more uniform particles are typically associated with faster solubilization and improved extraction yields, particularly for polysaccharides such as kappa-carrageenan [34]. *G. corneum* exhibited the largest particle size distribution (D50: 0.790 mm, D90: 1.348 mm), with a high mean particle size (0.821 mm) and broad variability (±0.446 mm). Larger particles reduce surface area availability, slowing down hydrocolloid diffusion and solubilization, which may affect final gel strength and agar extraction efficiency. In industrial-scale processing, controlling particle size is essential to optimize solubilization rates, minimize extraction time, and improve gel consistency. Studies have shown that reducing particle size prior to extraction can enhance mechanical properties and dissolution kinetics in hydrocolloid systems, making particle size optimization a key factor in process efficiency [36].

### 2.3. Texture Profile of Gels

**Figure 2:**
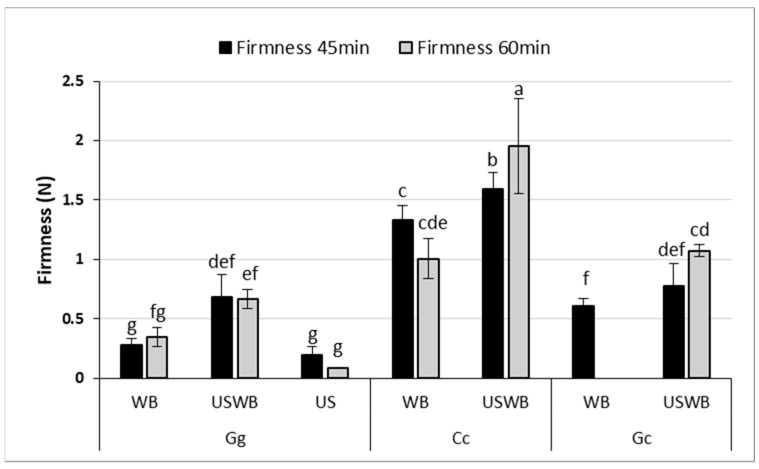
Firmness (N) of gels extracted from three seaweed species (*Gracilaria gracilis* (Gg), Chondrus crispus (Cc), and *Gelidium corneum* (Gc)) under three treatments: water bath (WB), ultrasound and water bath (USWB), and ultrasound (US). Firmness was measured after 45 min and 60 min of treatment. Note that US treatment was not conducted for *C. crispus* and *G. corneum*, nor was US at 45 min for *G. gracilis* and WB at 60 min for *G. corneum* evaluated. Different letters indicate statistically significant differences (*p* < 0.05).

Texture Profile Analysis (TPA) revealed significant differences in gel firmness and adhesiveness among seaweed species and treatments (Figure 2), demonstrating the impact of extraction methods on gel structure.

Firmness, which represents the resistance of the gel to deformation, varied considerably between the samples. *C. crispus* consistently formed the firmest gels, reinforcing the strong gel-forming properties of kappa-carrageenan. This effect was most pronounced in the 60 min USWB treatment, which yielded the highest firmness values. These findings align with previous studies showing that kappa-carrageenan produces highly elastic, stable gels with enhanced firmness [37,38].

For *G. gracilis*, gel firmness significantly improved with the USWB treatment compared to WB after 45 min of extraction, as indicated by statistical differences (*p* < 0.05). Although the high viscosity of *C. crispus* may have partially limited the effective propagation of ultrasound waves, the USWB treatment still resulted in a significant increase in gel firmness compared to WB alone. This superior performance can be attributed to the high viscosity and cohesiveness imparted by carrageenan, as reported in prior studies [38].

*G. gracilis* and *G. corneum* exhibited low adhesiveness across all treatments, with no significant differences between extraction times. This suggests that agar-based gels from these species may be unsuitable for applications requiring high cohesiveness, such as emulsion stabilization or dairy alternatives. In contrast, *C. crispus* exhibited markedly higher adhesiveness, exceeding 1.2 N.s across treatments. Interestingly, the hybrid USWB treatment did not significantly outperform the WB treatment alone in improving adhesiveness for any of the seaweed species. Ultrasound treatment slightly improved firmness, but did not enhance adhesiveness, particularly in *G. corneum* and *G. gracilis*. This suggests that ultrasound-induced polysaccharide fragmentation reduced molecular weight, limiting adhesive interactions while preserving gel structure [39,40]. *Gelidium corneum* showed the weakest gel strength, consistent with previous findings on its lower textural performance [41]. These findings underscore the need to optimize extraction conditions based on application requirements. The increase in firmness with USWB treatment suggests that ultrasound, when combined with controlled heating, enhances polysaccharide solubility and gel network formation. However, precise ultrasound intensity and exposure control are crucial, as excessive treatment can degrade polysaccharide chains, altering gel strength and viscosity [42,43].

Moreover, the low adhesiveness of *G. gracilis* and *G. corneum* suggests potential limitations for applications requiring cohesive gels, such as emulsion stabilization or textural enhancement in dairy alternatives [44]. In contrast, the high adhesiveness of *C. crispus* gels may be beneficial for formulations requiring robust gel structures, including plant-based desserts, pharmaceutical encapsulation, and biomedical hydrogels [45]. Understanding these textural variations is essential for selecting appropriate extraction methods based on the specific requirements of food and pharmaceutical applications.

### 2.4. Rheology Behavior of Gels

#### 2.4.1. Cooling and Maturation

**Figure 3:**
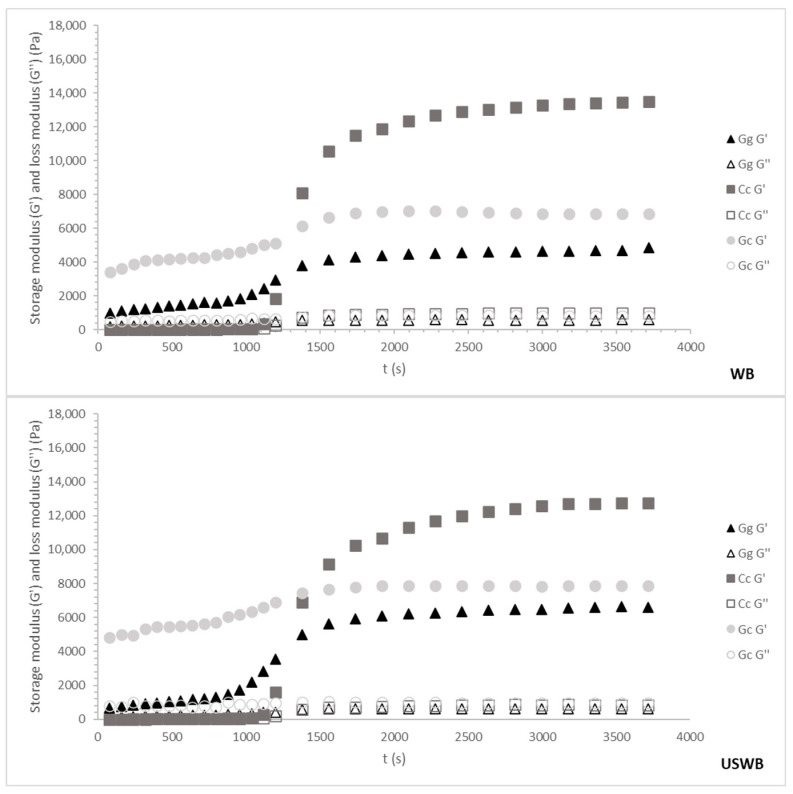
Cooling ramp and gel maturation for 45 min treatments from three seaweed species: *Gracilaria gracilis* (Gg), *Chondrus crispus* (Cc), and *Gelidium corneum* (Gc). The *G*′ (storage modulus) and *G*″ (loss modulus) values are plotted as the temperature decreases from 80 °C to 20 °C. WB: water bath, US: ultrasound, USWB: hybrid extraction (ultrasound and water bath combined).

**Figure 4:**
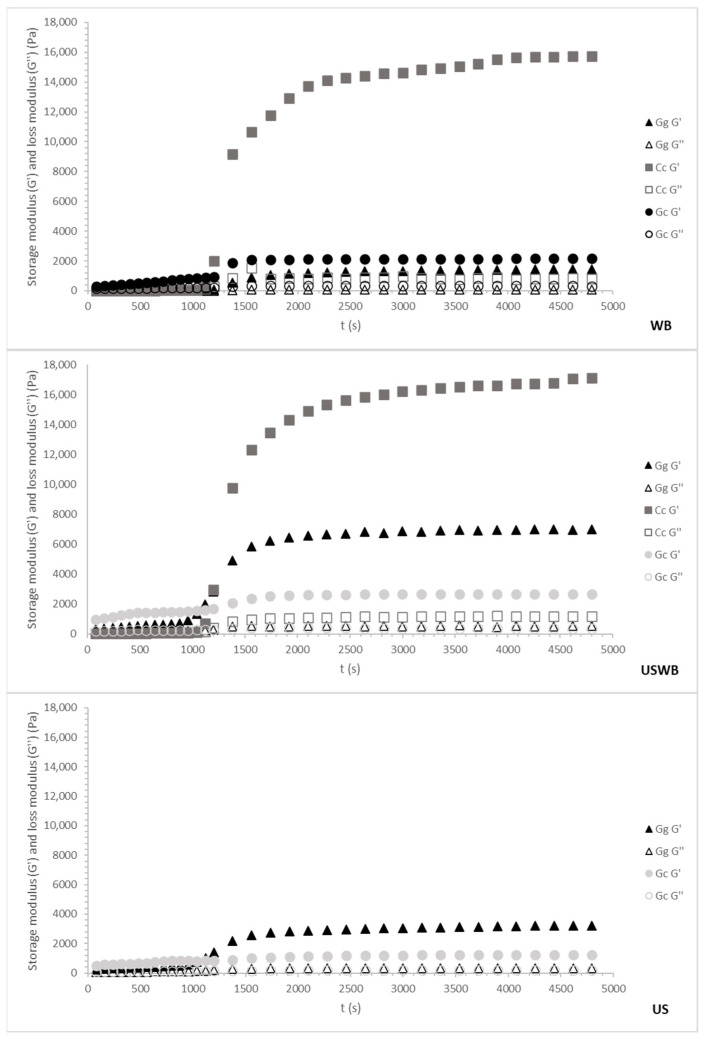
Cooling ramp and gel maturation for 60 min treatments from three seaweed species: *Gracilaria gracilis* (Gg), *Chondrus crispus* (Cc), and *Gelidium corneum* (Gc). The *G′* (storage modulus) and *G*″ (loss modulus) values are plotted as the temperature decreases from 80 °C to 20 °C. WB: water bath, US: ultrasound, USWB: hybrid extraction (ultrasound and water bath combined).

Figure 3 and Figure 4 illustrate the cooling and maturation behavior of the gels for the 45 min and 60 min treatments, respectively. These combined profiles provide insight into both gel formation during cooling and gel structural development over time.

Regardless of species or treatment, gelation occurred around 30 °C, aligning with known gelation temperatures for carrageenan and agar [46]. However, variations in G′ (storage modulus) among species and treatments revealed differences in gel strength and maturation kinetics, reflecting distinct hydrocolloid interactions.

45-min Treatments

For the 45 min treatments, *G. corneum* initially exhibited the highest G′ values, likely due to larger particle sizes contributing to immediate rigidity rather than true gel network formation. Throughout maturation, G′ remained stable, indicating early structural stabilization rather than progressive strengthening. This behavior aligns with *G. corneum’s* high agarose content, which promotes rapid gelation, but limited structural evolution over time [31].

*C. crispus* exhibited moderate G′ values during cooling, indicative of carrageenan’s slower gelation kinetics. This process involves the gradual formation of ionic interactions between sulphated polysaccharides and water molecules, leading to increased gel strength as temperature decreases [47]. During maturation, *C. crispus* exhibited a significant rise in G′, surpassing *G. corneum*. This late-stage stiffening suggests additional structural stabilization through secondary interactions such as hydrogen bonding and cross-linking between hydrocolloid molecules [37].

*G. gracilis* consistently showed the lowest overall G′ values during both cooling and maturation, reflecting its weaker gel-forming properties. This aligns with the characteristic softer gels of *Gracilaria*’s sp., as its agar is less robust compared to *Gelidium* agar or *Chondrus* carrageenan [48]. The maturation phase revealed only a slight increase in G′ values, indicating limited structural development over time.

Among treatments during 45 min, the WB method produced the strongest gels for all species, while the combination of ultrasound and water bath (USWB) slightly improved gel strength for *G. gracilis*. However, the ultrasound-only (US) treatment consistently resulted in the weakest gels, probably due to polysaccharide chain disruption interfering with the formation of a cohesive gel matrix [38].

60-min Treatments

The 60 min treatments showed trends like the 45 min treatments, but with notable differences in gel strength and structural stability (Figure 4).

During cooling, *G. corneum* again displayed the highest G′ values, confirming its robust gel-forming properties. However, the longer extraction time led to slightly reduced gel strength compared to the 45 min treatments, potentially due to the release of additional compounds that interfere with gelation. The maturation phase showed minimal changes, consistent with the early stabilization of the gel matrix.

*C. crispus* demonstrated a pronounced increase in G′ values during the maturation phase, further enhancing its gel strength beyond the cooling phase. This behavior suggests that extended extraction enhances the gelation properties of carrageenan due to the increased availability of sulphated polysaccharides that contribute to stronger secondary interactions [49].

*G. gracilis* showed the weakest gels during cooling and maturation. The 60 min extraction further reduced G′ values compared to the 45 min treatments, particularly under US and USWB methods. This reduction is due to extended ultrasound exposure causing a more pronounced breakdown of polysaccharide chains [50].

Overall, 45 min treatments produced stronger gels for the agarophyte species, with WB and USWB treatments being the most effective, while for the carragenophyte *C. crispus*, a 60 min extraction improved the structure of its gel. The combination of cooling and maturation profiles highlights the balance between gelation kinetics and structural development, with *C. crispus* exhibiting the most significant improvements during the maturation phase, while *G. corneum* maintained stable and strong gel matrices throughout both processes.

These findings underscore the importance of selecting the appropriate extraction method based on the intended application. The WB method consistently yielded stronger gels, making it particularly suitable for food applications requiring high gel strength, such as dairy or meat analogs [51]. The USWB method enhanced gelation in certain cases, but produced variables effects depending on the species, suggesting its potential for tailored applications. However, excessive ultrasound exposure led to the degradation of *G. gracilis* polysaccharides, emphasizing the need for precise processing control to preserve hydrocolloid integrity. Striking a balance between gel strength and processing efficiency is essential for optimizing hydrocolloid applications in food, pharmaceuticals, and biomedical formulations.

#### 2.4.2. Mechanical Structure

**Figure 5:**
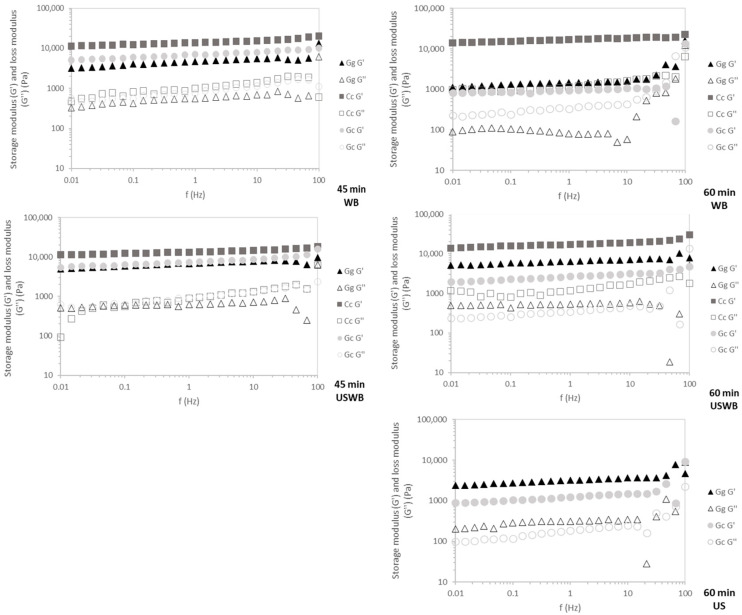
Mechanical spectra of the gels for 45 min and 60 min treatments showing *G*′ (storage modulus) and *G*″ (loss modulus) of three seaweed species: *Gracilaria gracilis* (Gg), *Chondrus crispus* (Cc), and *Gelidium corneum* (Gc). WB: water bath, US: ultrasound, USWB: hybrid extraction (ultrasound and water bath combined).

*C. crispus* exhibited the highest G′ values across all frequencies (Figure 5), confirming its stable carrageenan-based gel structure. This resilience indicates strong intermolecular interactions, which contribute to superior elasticity and mechanical strength [47].

*G. gracilis* and *G. corneum* displayed frequency-dependent behavior, characteristic of agar-based gels. During the 45 min treatments, G′ remained stable across tested frequencies, reflecting rapid gelation kinetics. However, in the 60 min extractions, increased frequency dependence—especially near 100 Hz—suggests that prolonged extraction may weaken gel network integrity, possibly due to polymer degradation or reduced intermolecular interactions, leading to greater variability in mechanical response [36].

WB and USWB for 45 min extractions yielded gels with the highest mechanical resilience, confirming that shorter extraction times preserve polymer integrity. Conversely, US treatments resulted in weaker gels with greater frequency dependence, particularly in *G. gracilis*, likely due to ultrasound-induced polymer fragmentation disrupting gel network formation [43].

*C. crispus* consistently produced the strongest and most stable gels across all treatments, while *G. corneum* and *G. gracilis* showed weaker and less stable gels. Shorter extraction times (45 min) and WB or USWB treatments were optimal for achieving resilient gels with high structural integrity.

These findings have direct implications for hydrocolloid formulation and processing. Carrageenan-based gels (*C. crispus*) exhibit superior mechanical resilience, making them suitable for applications requiring stable gel structures, such as dairy alternatives, processed meats, and functional foods [52,53]. Conversely, agar-based gels (*G. gracilis* and *G. corneum*) display higher frequency dependence, particularly after prolonged extraction, which may limit their use in high-shear applications like emulsified food systems [54]. However, their rapid gelation kinetics and moderate gel strength make them well-suited for confectionery plant-based gelatin replacements and controlled-release encapsulation technologies [54]. Optimizing extraction conditions is crucial to achieving the desired balance between yield, gel functionality, and mechanical resilience in hydrocolloid-based applications.

### 2.5. Carbohydrate Composition of WB Gels

*C. crispus*, *G. corneum*, and *G. gracilis* are valued for their distinct polysaccharide compositions, which provide gelling and stabilizing properties in industrial applications [55,56]. Among the three extraction methods tested, WB extraction was selected for further characterization due to its superior polysaccharide recovery efficiency. The selection was based on the ability of WB extraction to increase the total carbohydrate content in the extracts while preserving key structural components of hydrocolloids. Therefore, the carbohydrate composition analysis focused specifically on WB extracts to assess the effectiveness of this method in maintaining hydrocolloid integrity. Table 2 summarizes the carbohydrate content and composition of dried biomass and WB extracts, highlighting significant shifts post-extraction.

*C. crispus* primarily comprises galactose (74.1 mol%) and 3,6-anhydrogalactose (21.4 mol%), aligning with the literature values for kappa-carrageenan (65–75% galactose, 20–25% 3,6-AnGal) [49,50]. This composition reinforces its strong gelling properties, making *C. crispus* a key carrageenan source for food and pharmaceutical applications. Minor sugar residues, like xylose (1–2 mol%) in our data, align with findings that small amounts of these sugars can contribute to enhanced gel elasticity and strength [21]. WB extraction increased 3,6-AnGal content to 25.2 mol%, while galactose slightly decreased to 67.9 mol%. This shift suggests that WB selectively solubilizes sulfated galactans while retaining anhydrogalactose-rich fractions, likely due to the higher solubility and extraction efficiency of 3,6-AnGal under controlled thermal conditions [21,57]. The slight reduction in galactose may reflect its partial hydrolysis or diffusion into the extraction medium rather than an actual compositional loss.

*G. gracilis* biomass contained 33.2 mol% 3,6-AnGal, 50.7 mol% galactose, and 8.8 mol% 6-methylgalactose, consistent with polysaccharide profiles reported for *Gracilaria* species. Studies by Martin et al. [58] and Rodríguez et al. [59] describe 3,6-AnGal levels ranging from 40 to 45 mol% and galactose levels between 38 and 42 mol%, highlighting environmental influences on agar composition. The presence of 6-methylgalactose enhances solubility and flexibility, making *G. gracilis* ideal for applications requiring pliable gel formulations, such as confectionery and biomedical hydrogels. After WB treatment, 3,6-AnGal decreased to 25.7 mol%, while galactose increased to 60.0 mol%, maintaining agaropectin content and confirming the extraction method’s efficiency in retaining essential polysaccharide properties.

For *G. corneum*, biomass contained 31.1 mol% of 3,6-AnGal and 53.3 mol% of galactose, aligning with the literature for *Gelidium spp*., where 3,6-AnGal ranges from 30 to 35 mol% and galactose from 50 to 55 mol% [60]. *G. corneum* produces high-quality agar with exceptional clarity and gel strength, valuable for microbiological and biotechnological applications. Minor sugars such as xylose (1–2 mol%) are also commonly found in *Gelidium*, contributing to its structural stability [61]. WB extraction resulted in a significant decrease (*p* < 0.05) in 3,6-AnGal to 24.6 mol%, while the increase in galactose to 55.6 mol% was not statistically significant (*p* > 0.05). This suggests that WB extraction may selectively affect certain polysaccharide components, leading to compositional shifts that impact gelation properties, while other structural elements remain stable.

WB extraction modified the balance between 3,6-AnGal and galactose across all species while preserving essential hydrocolloid properties. *C. crispus* retained carrageenan integrity, *G. corneum* maintained its high-quality agar, and *G. gracilis* preserved its agaropectin content, demonstrating the effectiveness of WB extraction in producing functionally stable hydrocolloids for industrial applications [49,62,63].

### 2.6. Antioxidant Activity and Total Phenolic Content of Gels

As expected, the crude seaweed biomass exhibited the highest antioxidant activity across all species, with *G. gracilis* showing the highest DPPH value (16.6 µmol EQ Trolox/g), followed by *G. corneum* (11.7 µmol EQ Trolox/g) and *C. crispus* (10.5 µmol EQ Trolox/g) (Table 3). After extraction, this trend remained consistent, with *C. crispus* retaining the lowest antioxidant activity in USWB- and US-treated gels. WB-treated gels retained higher antioxidant activity than US- and USWB-treated gels, particularly in *G. gracilis* and *C. crispus*, suggesting greater thermal stability of their antioxidants. However, *G. corneum* exhibited a 49.3% decrease in DPPH values following WB treatment, indicating a higher sensitivity to thermal degradation. Conversely, US treatment significantly reduced antioxidant activity across all species, likely due to oxidative degradation from cavitation effects. Prolonged ultrasound exposure can disrupt antioxidant structures, reducing their efficacy [64]. Similarly, hybrid USWB treatment resulted in lower antioxidant potential than WB, highlighting the need for parameter optimization to minimize antioxidant losses [65]. The FRAP assay results mirrored the DPPH trends, with *G. gracilis* displaying the highest reducing power, followed by *G. corneum* and *C. crispus*. WB-treated gels retained relatively stable FRAP values, particularly in *G. corneum*, suggesting the better preservation of reducing agents compared to *G. gracilis* and *C. crispus*. However, US-treated gels consistently showed the lowest FRAP values, reinforcing the detrimental impact of ultrasound on antioxidant integrity.

The total phenolic content varied significantly among crude biomass samples, with *C. crispus* containing the highest levels (9.1 µmol EQ Gallic acid/g) and *G. corneum* the lowest (1.7 µmol EQ Gallic acid/g). These results suggest that *G. corneum* has an inherently lower phenolic composition. After extraction, WB-treated gels showed moderate reductions in phenolic content, with *G. corneum* experiencing the least variation, indicating higher thermal stability. Conversely, both US and hybrid USWB treatments caused substantial phenolic losses, with US-treated gels displaying the lowest retention values.

Overall, the DPPH and FRAP assays demonstrated that WB treatment preserved antioxidants and phenolic compounds more effectively than ultrasound-based methods. *C. crispus* was particularly affected, showing the lowest retention levels post-treatment. Given that phenolic compounds, especially hydroxyl-rich ones, are prone to oxidative degradation, optimizing ultrasound parameters is essential to preserving antioxidant integrity in seaweed-derived gels [66]. These findings reinforce the impact of processing conditions on bioactive compound retention, consistent with prior research on macroalgae-enriched gluten-free pasta, where processing methods influenced antioxidant stability and nutritional quality [4,67]. WB treatment preserved phenolic compounds and antioxidant activity more effectively than ultrasound-based methods, making it ideal for applications requiring high bioactive retention, such as functional foods, nutraceuticals, and antioxidant-enriched formulations.

### 2.7. Color of Gels

Color differences were analyzed in both untreated biomass and extracted gels. Biomass samples exhibited lower L* values, indicating darker coloration compared to extracted gels. WB-treated gels appeared as the darkest, likely due to high-temperature pigment degradation (90 °C), whereas US-treated gels were significantly lighter (Table 4). Thermal processing destabilizes pigments, such as chlorophyll, phycobiliproteins, and carotenoids, leading to reduced color intensity [68,69].

For the a* parameter, *G. corneum* exhibited the highest values, particularly after WB treatment, indicating greater pigment degradation. In contrast, US treatment better preserved pigmentation, likely due to milder processing conditions. Ultrasound-assisted extraction has been shown to protect pigments by minimizing thermal exposure [18].

For the b* parameter, crude samples—particularly *C. crispus*—exhibited high values, indicating greater yellowness. WB treatment further increased b*, likely due to pigment breakdown and the formation of yellowish-brown compounds under high temperatures. In contrast, US treatment resulted in lower b* values, suggesting improved pigment preservation, consistent with studies highlighting ultrasound’s protective effect on natural pigments [70].

Δ*E* values, representing overall color difference, were all above 5, indicating visible color changes between treatments and crude samples. Both WB and USWB treatments caused larger Δ*E* values in *G. gracilis* and *G. corneum*, confirming that thermal and mechanical processing significantly altered color. These findings highlight the impact of processing conditions on the visual properties of seaweed-derived gels, with thermal degradation playing a major role in pigment alteration [71].

Color is a critical factor in consumer perception and product marketability. The superior color stability of US-treated gels suggests that ultrasound-assisted extraction may be advantageous for preserving the natural appearance in food and cosmetic applications. In contrast, WB-treated gels may require formulation adjustments to counteract thermal-induced darkening, particularly in visually sensitive products. These findings highlight the importance of optimizing processing parameters to balance pigment retention with bioactive compound extraction, ensuring both visual appeal and functional quality in the final product [68,69].

## 3. Conclusions

This study highlights the potential of three red macroalgae species as sustainable hydrocolloid sources for food applications. The extraction method and duration significantly influenced the physicochemical and functional properties of the resulting gels. *Chondrus crispus* produced the firmest gels, particularly under WB and USWB treatments, due to its high carrageenan content and superior gel-forming ability. In contrast, *Gracilaria gracilis* and *Gelidium corneum* formed weaker gels, reflecting their agar composition. Additionally, WB extraction best preserved antioxidant activity, while US and USWB treatments led to significant bioactive compound losses, likely due to ultrasonic cavitation. These findings emphasize the need to optimize extraction parameters to balance hydrocolloid yield with bioactive compound retention.

This study also identified limitations related to sample preparation and treatment efficacy. The high viscosity of *C. crispus* hindered ultrasound propagation, while the large particle size of *G. corneum* caused sedimentation issues during gel formation. Consequently, WB and USWB treatments at 45 min proved the most effective for producing gels with desirable textural and rheological properties. These insights provide a foundation for further research into macroalgae-derived hydrocolloids in food systems requiring specific functional properties. Future studies should explore additional macroalgae species and refine extraction conditions to enhance hydrocolloid yield, functionality, and sensory attributes.

This study highlights key insights into macroalgae-derived hydrocolloids, but acknowledges its limitations, including ultrasound inefficiency due to *C. crispus* viscosity and sedimentation issues with *G. corneum*. Optimizing extraction parameters and exploring additional macroalgae species could improve yield and applicability. Future research should assess scalability, cost-effectiveness, and environmental impact to ensure feasibility for industrial clean-label hydrocolloid production.

Beyond technical optimization, the sustainability and renewability of macroalgae reinforce their potential as eco-friendly hydrocolloid alternatives. Their incorporation into food and pharmaceutical formulations aligns with the growing demand for clean-label ingredients, reducing reliance on synthetic or land-intensive hydrocolloid sources. Thus, macroalgae represent a promising avenue for the development of innovative, sustainable ingredients for the food and pharmaceutical industries.

## 4. Materials and Methods

### 4.1. Seaweed Samples

The macronutrient composition of *Chondrus crispus*, *Gracilaria gracilis*, and *Gelidium corneum* is presented in Table 5. The values for *C. crispus* and *G. gracilis* were provided by AlgaPlus (Aveiro, Portugal) and correspond specifically to the batches used in this study. The *G. corneum* samples were obtained from Iberagar (Barreiro, Portugal); however, no specific compositional data were available for these batches. According to Iberagar, their estimations are based on the literature data, particularly studies that analyzed *G. corneum* samples from similar sources. For this study, the values reported by Mouga and Fernandes [72] were used as a reference, as this work includes data relevant to *G. corneum* biomass supplied by Iberagar. Table 5 summarizes the macronutrient composition of these macroalgae, which is relevant for understanding their hydrocolloid content.

### 4.2. Particle Size Analysis by Laser Diffraction

The particle size distribution of the seaweed powders was analyzed in dry mode using the Partica LA-960 laser diffraction analyzer (Horiba, New Delhi, India). The optical refractive index was set to 1.450, with an imaginary component of 1.000. Data processing followed Mie scattering theory using the LA-960 software (version V2-885). Particle size was reported as D10 (diameter below which 10% of particles fall), D50 (median particle diameter), and D90 (diameter below which 90% of particles fall). All analyses were performed in triplicate.

### 4.3. Extraction Methodologies

Each macroalgal species was hydrated at a 1:15 ratio (13.34 g seaweed powder per 200 mL distilled water) in 250 mL beakers (70 mm diameter) to standardize conditions across samples. Hydration was conducted on a magnetic stirring plate (AREX, VELP Scientifica, Usmate Velate, Italy) at 20 °C for 30 min to ensure uniform mixing and water absorption, providing a homogeneous mixture for extraction.

After hydration, seaweed samples underwent three extraction treatments: water bath (WB), ultrasound (US), and a combination of both (USWB). Extraction durations of 45 and 60 min were tested to assess their impact on yield and efficiency. Preliminary tests at 15 and 30 min showed suboptimal polysaccharide recovery, leading to the selection of longer durations. Further increases beyond 60 min were considered impractical for industrial applications due to diminishing extraction efficiency. All treatments (WB, US, and USWB) were performed in triplicate to ensure reproducibility.

#### 4.3.1. Water Bath Extraction (WB)

Water bath extraction was performed using a Thermo Scientific Precision 2871 Reciprocating Water Bath (Waltham, MA, USA) preheated to 90 °C. Hydrated seaweed samples were submerged for either 45 or 60 min, depending on the treatment duration.

#### 4.3.2. Ultrasound-Assisted Extraction (US)

Ultrasound-assisted extraction (US) was performed using a UP200Ht Handheld Ultrasonic Homogenizer (Hielscher, Teltow, Germany) at 25 kHz and 150 W. A 14 mm diameter probe was fully submerged in the hydrated seaweed solution, with sonication lasting 45 or 60 min. To prevent overheating, the temperature was maintained at 60 °C using an ice bath.

#### 4.3.3. Hybrid Extraction (USWB)

The hybrid extraction method (USWB) combined 15 min of ultrasound treatment (as described above) with either 45 or 60 min of water bath extraction at 90 °C using a Thermo Scientific Precision Water Bath (Waltham, MA, USA). This approach was designed to optimize extraction efficiency by leveraging the benefits of both techniques.

### 4.4. Limitations and Adjustments

During the hydration process, the *C. crispus* sample exhibited excessive thickening, which rendered the application of ultrasound treatment ineffective. The high viscosity hindered the propagation of ultrasound waves, making it impossible to conduct this treatment for this species. For *G. corneum*, the large particle size caused significant settling during the gel formation process, resulting in inconsistencies in the measurements obtained using the texturometer and rheometer, as the sampled portions displayed varying characteristics, reflecting the gel’s heterogeneous nature. Ultrasound treatment for 45 min did not achieve effective extraction across all samples. As a result, standalone 45 min ultrasound trials were excluded, and the focus shifted to WB and USWB treatments. However, 60 min US treatments were retained for a more comprehensive evaluation of ultrasound effects.

### 4.5. Data Collection

#### 4.5.1. Texture Profile Analysis

Texture Profile Analysis (TPA) was performed to assess the mechanical properties of seaweed-derived gels using a TA.XT Plus texture analyzer (Stable Micro Systems, Godalming, Surrey, UK) with a 5 kg load cell. A cylindrical probe (P/10: DIA Cylinder Delrin, 10 mm diameter, 35 mm height, 78.54 mm^2^ contact area) was used for sample deformation. All tests were conducted at a controlled temperature of 20 ± 1 °C.

The analysis was carried out in penetration mode at a speed of 1 mm/s, followed by a post-test speed of 5 mm/s. A waiting time of 5 s was applied between the two cycles. A trigger force of 0.030 N was used to initiate contact with the sample. Key textural parameters were recorded, including firmness and adhesiveness, providing comprehensive insights into the gel’s macrostructural properties.

#### 4.5.2. Rheology Analysis

Rheological analysis was conducted using a MARS III controlled-stress rheometer (Thermo Scientific Haake, Karlsruhe, Germany) with an air compression system (Eheim Professional 3). Temperature control was maintained via a Peltier system. A parallel plate geometry (PP35Ti, 35 mm diameter, serrated surfaces) was used to prevent sample slippage, with a 1 mm gap to ensure uniform sample distribution and accurate measurements. The seaweed solutions were transferred to the rheometer immediately after the treatment phase, allowing for the cooling and gel maturation processes, as well as mechanical spectrum analysis, to occur entirely within the equipment. After placing the sample on the plate, liquid paraffin was applied to the edges to minimize water loss due to evaporation. Prior to these oscillatory tests, stress sweep tests were conducted at a constant oscillation frequency of 1 Hz to determine the linear viscoelastic region (LVER). Samples were allowed to rest for 10 min before analysis to eliminate any stress history. Each measurement was performed in triplicate to ensure reliability and reproducibility.

##### Temperature Sweep Test (Cooling Ramp)

The temperature sweep test assessed gelation behavior during cooling. Samples were preheated to 80 ± 0.5 °C for 60 s to ensure uniform starting conditions, then cooled to 20 ± 0.5 °C at a controlled rate of 3 °C/min. The viscoelastic properties, storage modulus (G′), and loss modulus (G″) were continuously monitored. A constant oscillation frequency of 1 Hz and a stress of 1 Pa ensured measurements remained within the LVER for accurate gelation dynamics assessment.

##### Time Sweep Test (Gel Maturation)

The time sweep test assessed gel maturation, stability, and structural evolution over 60 min at a constant temperature of 20 ± 0.5 °C. A fixed oscillation frequency of 1 Hz and a stress of 5 Pa ensured measurements remained within the LVER. Viscoelastic moduli (G′ and G″) were recorded over time to monitor the progression of gel strength and stability.

##### Frequency Sweep Test

The frequency sweep test evaluated the viscoelastic behavior of matured seaweed gels across oscillation frequencies ranging from 0.01 to 100 Hz. Measurements were conducted at 20 ± 0.5 °C with an applied stress of 5 Pa, ensuring that samples remained within the LVER. Viscoelastic moduli (G′ and G″) were recorded, providing insights into the gels’ elastic (solid-like) and viscous (liquid-like) properties.

#### 4.5.3. Carbohydrate Analysis

Carbohydrate analysis focused on WB-treated samples (60 min), as this condition yielded the most promising results based on the texture and rheological analyses. Prior to analysis, biomass and WB extracts were dialyzed (12–14 kDa molecular weight cutoff) at 4 °C to remove low-molecular-weight compounds. Polysaccharide content and composition were determined using reductive hydrolysis followed by acetylation, based on Stevenson and Furneaux [73]. The method employed borane-4-methylmorpholine complex (MMB) as a reducing agent, enabling simultaneous hydrolysis and reduction while preserving 3,6-anhydrogalactose (3,6-AnGal) residues from acidic degradation.

The resulting alditols were acetylated and analyzed via gas chromatography–mass spectrometry (GC-MS, Shimadzu GC–MS QP2010, Shimadzu Corporation, Kyoto, Japan) with a ZB-5HT capillary column (30 m × 0.25 mm i.d., 0.25 μm film thickness, J&W Scientific, Folsom, CA, USA). Samples were injected in split mode at 250 °C. The oven temperature program was set to 140 °C, increased to 180 °C at 5 °C/min (holding for 1 min), then ramped to 280 °C at 10 °C/min. Helium was used as the carrier gas (32.1 mL/min). This method enabled precise sugar quantification, offering detailed insights into polysaccharide structural composition.

#### 4.5.4. Antioxidant Analysis

Antioxidant capacity was assessed using three complementary assays: DPPH (2,2-diphenyl-1-picrylhydrazyl), FRAP (Ferric Reducing Antioxidant Power), and Total Phenolic Content (TPC) via the Folin–Ciocalteu method [73,74,75]. These assays evaluate different antioxidant mechanisms, offering a comprehensive profile of extract antioxidative properties. All analyses were conducted using a CLARIOstar Plus microplate reader (BMG LABTECH, Ortenberg, Germany).

Prior to analysis, the seaweed extracts underwent methanol-based extraction using a 1:4 ratio of sample to methanol, optimized in preliminary experiments. The extraction was carried out overnight to ensure the maximum recovery of bioactive compounds. Methanolic extracts were then used for the analysis, with untreated (crude) samples serving as a reference. A 1% ascorbic acid solution was included as a positive control to benchmark antioxidant activity.

##### DPPH Assay

A DPPH reagent solution was prepared, and absorbance was measured at 517 nm. Absorbance was maintained above 0.7, with distilled water as the blank. In 2 mL tubes, 20 µL of sample extract was mixed with 180 µL of DPPH reagent, incubated in darkness for 30 min at room temperature, and absorbance was recorded.

##### FRAP Assay

The FRAP reagent was prepared by mixing 2,4,6-tripyridyl-s-triazine (TPTZ), FeCl3, and acetate buffer (1:1:10 ratio). Absorbance was measured at 593 nm, with methanol as the blank. For the assay, 25 µL of each extract was pipetted into 2 mL tubes, mixed with 175 µL of FRAP reagent, and incubated at 37 °C for 15 min. Samples were further incubated in darkness for 30 min before absorbance readings.

##### Total Phenolic Content (Folin–Ciocalteu Assay)

The total phenolic content (TPC) was quantified using the Folin–Ciocalteu method, with absorbance measured at 760 nm. Methanol (MeOH) was used as the blank. For each assay, 20 µL of the sample extract was added to 2 mL tubes, followed by 100 µL of Folin–Ciocalteu reagent and 80 µL of distilled water. The mixture was incubated at room temperature for 30 min to allow for the reaction to stabilize, and the absorbance readings were then recorded.

#### 4.5.5. Color Analysis

Color measurements were conducted on both untreated biomass and extracted gel samples using a Minolta CR-400 colorimeter (Konica Minolta Sensing, Osaka, Japan) based on the CIELab color system. This system quantifies color attributes as L* (lightness), a* (green to red), and b* (blue to yellow). The total color difference (Δ*E*) between samples was calculated using Equation (1), where *L*_1_, *a*_1_, and *b*_1_ correspond to the color values of the reference sample, and *L*_2_, *a*_2_, and *b*_2_ correspond to the color values of the analyzed sample.

(1) $$\Delta E=\sqrt{\left\{{\left(L_{2}-L_{1}\right)}^{2}+{\left(a_{2}-a_{1}\right)}^{2}+{\left(b_{2}-b_{1}\right)}^{2}\right\}}$$

For the measurements, the samples were placed under the colorimeter sensor, and each sample was measured in triplicate. The average values of L*, a*, and b* were recorded and used to evaluate the color attributes of the developed samples. This method provides a reliable and consistent evaluation of color properties, which is essential for understanding consumer acceptance.

#### 4.5.6. Experimental Workflow Overview

**Figure 6:**
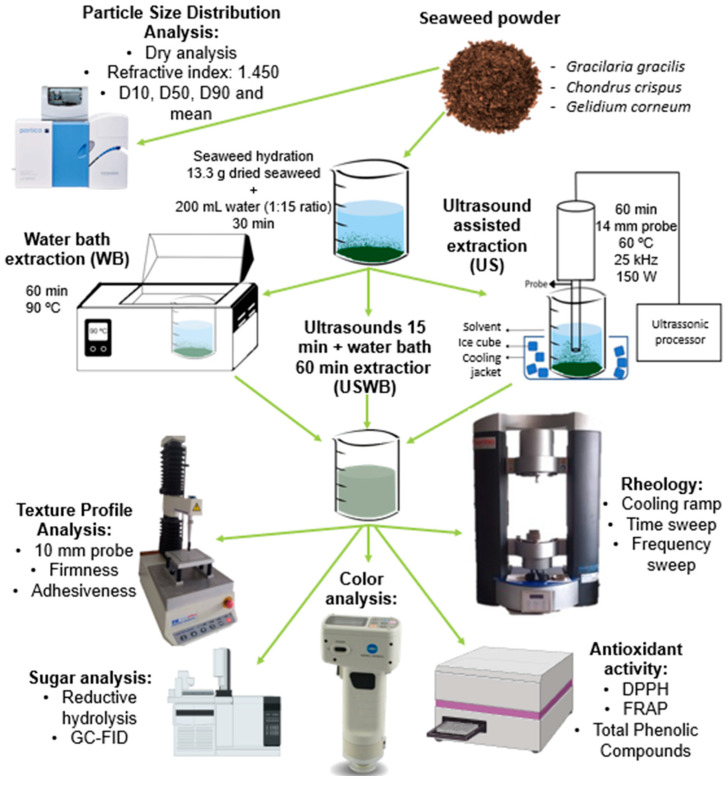
Schematic representation of the extraction methods and analytical techniques used in this study, including particle size analysis, texture profile, rheology, carbohydrate composition, antioxidant activity, and color evaluation.

Figure 6 presents a schematic overview of the experimental workflow, summarizing the extraction processes, instrumental analyses, and key methodologies used in this study. This visual representation aims to facilitate understanding of the experimental design and the interactions between various analytical techniques.

#### 4.5.7. Statistical Analysis

All data were analyzed using Microsoft Excel and GraphPad Prism 9.5.1 software for statistical processing. Results are presented as the mean ± standard deviation (SD) from triplicate measurements for each analysis. Statistical significance was determined by performing a one-way analysis of variance (ANOVA) followed by Tukey’s multiple comparisons test to assess significant differences between sample means (*p* < 0.05).

## Figures and Tables

**Table 1:** Particle size distribution of *G. gracilis*, *C. crispus*, and *G. corneum* (in mm).

Sample	D10 (mm)	D50 (mm)	D90 (mm)	Mean (mm)
*Gracilaria gracilis*	0.288 ± 0.120 ^a^	0.550 ± 0.305 ^B^	1.108 ± 0.410 ^2^	0.635 ± 0.353 ^β^
*Chondrus crispus*	0.052 ± 0.045 ^b^	0.329 ± 0.137 ^C^	0.568 ± 0.238 ^3^	0.330 ± 0.193 ^γ^
*Gelidium corneum*	0.252 ± 0.186 ^a^	0.790 ± 0.350 ^A^	1.348 ± 0.596 ^1^	0.821 ± 0.446 ^α^

D10, D50, and D90 represent the particle size diameters below in which 10%, 50%, and 90% of the particles, respectively, are distributed. Mean values are presented as mean ± standard deviation (n = 3). Different superscript letters, numbers and symbols within the same column indicate significant differences between samples (*p* < 0.05, Tukey test).

**Table 2:** Carbohydrate composition (mol%) and total carbohydrate content (%*w*/*w* DW) of the dialyzed biomass and dialyzed water bath (WB) extracts obtained from *Chondrus crispus*, *Gelidium corneum*, and *Gracilaria gracilis* during 60 min extraction.

		Monosaccharides (mol%)								Total Monosaccharides (%*w*/*w* DW)
	Sample	3,6AnGal	Xyl	6-MethylGal	2-MethylGal	4-MehylGal	Man	Gal	Glc	
Biomass	*C. crispus*	21.4 ± 2.1 **	1.8 ± 0.2 *	-	-	tr	tr	74.1 ± 1.7	2.7 ± 0.9 **	36.8 ± 2.2 **
	* G. gracilis *	33.2 ± 0.9	1.6 ± 0.0 *	8.8 ± 2.1 **	tr	tr	tr	50.7 ± 1.1 **	4.9 ± 1.5 **	46.1 ± 2.4 **
	* G. corneum *	31.1 ± 0.4 *	1.6 ± 0.2 *	tr	tr	tr	tr	53.3 ± 2.9 **	14.1 ± 3.1	53.8% ± 4.9 **
WB	*C. crispus*	25.2 ± 0.8 *	1.5 ± 0.1 **	-	-	tr	tr	67.9 ± 1.2 **	5.2 ± 0.4 **	43.1 ± 1.8 **
	* G. gracilis *	25.7 ± 2.6 *	2.1 ± 0.1 *	8.9 ± 1.3 **	tr	1.2 ± 0.2 **	tr	60.0 ± 1.5 **	2.1 ± 0.2 **	65.3 ± 1.5 *
	* G. corneum *	24.6 ± 4.0 *	2.5 ± 0.6 *	1.9 ± 0.3 **	1.3 ± 0.0 **	1.0 ± 0.0 **	tr	55.6 ± 2.3 **	13.1 ± 5.6	63.4 ± 4.3 *

Data are expressed as mean ± standard deviation (n = 3). tr = trace amounts (<1 mol%), - = undetected. A single asterisk (*) indicates a significant difference (*p* < 0.05); double asterisks (**) indicate a highly significant difference (*p* < 0.05). Abbreviations: 3,6AnGal—3,6-anhydrogalactose, Xyl—xylose, 6-MethylGal—6-methylgalactose, 2-MethylGal—2-methylgalactose, 4-MethylGal—4-methylgalactose, Man—mannose, Gal—galactose, and Glc—glucose. Tr—trace amounts (<1 mol%). and “-”—undetected components.

**Table 3:** Antioxidant activity (DPPH, FRAP) and total phenolic compounds in crude biomass and extracted gel samples of *Gracilaria gracilis* (Gg), *Chondrus crispus* (Cc), and *Gelidium corneum* (Gc) following different extraction treatments (WB, USWB, US). Results are expressed in µmol EQ Trolox/g for DPPH and FRAP, and µmol EQ Gallic acid/g for total phenolics. Ascorbic acid was used as a reference antioxidant. Different letters indicate statistically significant differences (*p* < 0.05).

Samples		DPPH (µmol EQ Trolox/g of Sample)	FRAP (µmol EQ Trolox/g of Sample)	Total Phenolic Compounds (µmol EQ Gallic Acid/g of Sample)
Biomass	Gg	22.4 ± 1.05 ^b^	16.6 ± 2.25 ^a^	7.78 ± 0.86 ^c^
	Cc	16.9 ± 1.25 ^c^	10.5 ± 2.30 ^c^	9.19 ± 0.04 ^b^
	Gc	20.3 ± 3.06 ^b^	11.7 ± 0.88 ^b^	1.73 ± 0.02 ^f^
WB	Gg	20.5 ± 2.53 ^b^	2.53 ± 0.171 ^e^	2.36 ± 0.36 ^e^
	Cc	14.5 ± 2.79 ^d^	2.62 ± 1.40 ^e^	1.46 ± 0.13 ^g^
	Gc	10.3 ± 1.72 ^e^	2.81 ± 0.13 ^d^	1.40 ± 0.20 ^g^
USWB	Gg	6.01 ± 0.42 ^f^	3.15 ± 0.23 ^d^	2.70 ± 0.28 ^d^
	Cc	0.32 ± 0.22 ^g^	2.69 ± 0.39 ^e^	2.17 ± 0.14 ^e^
	Gc	0.85 ± 0.23 ^g^	3.23 ± 0.19 ^d^	0.98 ± 0.08 ^h^
US	Gg	5.92 ± 2.20 ^f^	2.27 ± 0.12 ^ef^	1.68 ± 0.16 ^f^
	Gc	1.00 ± 0.14 ^g^	1.84 ± 0.17 ^f^	0.53 ± 0.39 ^h^
Ascorbic acid		9344 ± 106.2 ^a^	Overflow	4675 ± 56.6 ^a^

**Table 4:** Color parameters (L*, a*, b*) and overall color difference (Δ*E*) of *Gracilaria gracilis* (Gg), *Chondrus crispus* (Cc), and *Gelidium corneum* (Gc) under different treatments. Values represent the mean ± standard deviation. Significant differences between treatments were determined using ANOVA (*p* < 0.05), and statistically significant differences are indicated by different letters. WB: water bath, US: ultrasound, USWB: hybrid extraction (ultrasound and water bath combined).

Samples		L*	a*	b*	Δ*E*
Biomass	Gg	44.5 ± 2.10 ^a^	−0.43 ± 0.03 ^d^	5.07 ± 0.51 ^d^	
	Cc	45.6 ± 0.85 ^a^	0.29 ± 0.26 ^f^	8.42 ± 0.30 ^a^	
	Gc	37.0 ± 0.32 ^b^	8.29 ± 0.24 ^a^	2.57 ± 0.15 ^e^	
WB	Gg	21.4 ± 0.16 ^θ^	1.71 ± 0.33 ^d^	5.51 ± 0.27 ^c^	24.4
	Cc	22.1 ± 0.54 ^g^	1.26 ± 0.17 ^d^	7.07 ± 0.26 ^c^	22.5
	Gc	21.2 ± 0.11 ᶿ	2.81 ± 0.40 ^c^	5.38 ± 0.24 ^d^	16.9
USWB	Gg	23.2 ± 0.34 ^d^	−0.29 ± 0.49 ^f^	7.97 ± 0.46 ^a^	22.4
	Cc	26.0 ± 0.37 ^d^	−0.53 ± 0.20 ^f^	8.54 ± 0.17 ^b^	18.8
	Gc	20.7 ± 0.57 ^e^	1.44 ± 0.08 ^d^	3.31 ± 0.57 ^e^	17.7
US	Gg	27.6 ± 0.37 ^c^	0.63 ± 0.23 ^g^	9.43 ± 0.09 ^a^	17.4
	Gc	20.6 ± 0.52 ^e^	7.31 ± 0.06 ^b^	3.07 ± 0.12 ^e^	16.4

**Table 5:** Macronutrient composition of *Chondrus crispus*, *Gracilaria gracilis*, and *Gelidium corneum* per 100 g of dried product.

	* Chondrus crispus *	* Gracilaria gracilis *	* Gelidium corneum *
Energy (kcal/kJ)	301/1261	282/1183	208/874
Total fat (g)	1.00	1.20	2.10
Carbohydrates (g)	55.80	47.00	33.30
Protein (g)	17.30	21.00	14.19

## Data Availability

The original contributions presented in the study are included in the article; further inquiries can be directed to the corresponding author.
